# Utility and Future Perspectives of Circulating Tumor DNA Analysis in Non-Small Cell Lung Cancer Patients in the Era of Perioperative Chemo-Immunotherapy

**DOI:** 10.3390/cells14171312

**Published:** 2025-08-24

**Authors:** Shuta Ohara, Kenichi Suda, Yasuhiro Tsutani

**Affiliations:** 1 Division of Thoracic Surgery, Department of Surgery, Kindai University Faculty of Medicine, 377-2 Ohno-Higashi, Osaka-Sayama 589-8511, Japan; 154148@med.kindai.ac.jp (S.O.); yatsutani@msn.com (Y.T.); 2 Division of Thoracic Surgery, Izumi City General Hospital, 4-5-1 Wake-cho, Izumi 594-0073, Japan

**Keywords:** circulating tumor DNA (ctDNA), liquid biopsy, perioperative treatment, neoadjuvant treatment, non-small cell lung cancer (NSCLC), pathological response, prognostic factor

## Abstract

Perioperative/neoadjuvant chemo-immunotherapy is a standard treatment for patients with resectable non-small cell lung cancer (NSCLC). However, several key clinical questions remain unresolved, including the monitoring of tumor response during neoadjuvant treatment, detection of residual disease after neoadjuvant treatment or after surgery, stratification of recurrence risk, and earlier detection of disease recurrence. Circulating tumor DNA (ctDNA) has emerged as a promising biomarker to address these challenges. Data from several recent clinical trials of perioperative/neoadjuvant chemo-immunotherapy demonstrated that ctDNA clearance before surgery was associated with higher rates of major pathological response. Additionally, landmark ctDNA positivity after surgery identified patients at high risk of disease recurrence, and longitudinal ctDNA monitoring enabled earlier detection of recurrence compared with radiographic surveillance. Several ongoing trials are incorporating ctDNA as a biomarker to guide treatment decisions, including optimizing the duration of neoadjuvant therapy, evaluating the need for surgery, and tailoring adjuvant strategies. These trials, together with further development of ctDNA detection technologies, will clarify the role of ctDNA analysis in refining perioperative treatment strategies and may ultimately enable individualized care in patients with resectable NSCLC. In this review, we discuss the current research data on ctDNA analysis in NSCLC in this era of perioperative chemo-immunotherapy.

## 1. Introduction

The clinical application of adjuvant immunotherapy [1,2] and neoadjuvant/perioperative chemo-immunotherapy [3,4,5] has expanded the treatment options for patients with resectable but locally advanced non-small cell lung cancer (NSCLC). At present, there are no definite criteria to determine the appropriate treatment for each patient regarding upfront surgery followed by adjuvant immunotherapy or neoadjuvant chemo-immunotherapy before pulmonary resection. In general, neoadjuvant chemo-immunotherapy should be chosen for patients with more advanced disease who may have a higher risk of undetectable micro-metastases, with the purpose of inducing early systemic treatment. Furthermore, for patients with “marginally” resectable NSCLC, it is anticipated that the surgical time will be shortened and the likelihood of complete resection will increase if the tumor shrinks by neoadjuvant chemo-immunotherapy. Currently, neoadjuvant nivolumab plus chemotherapy (the CheckMate-816 regimen), neoadjuvant pembrolizumab plus chemotherapy followed by adjuvant pembrolizumab (the KEYNOTE-671 regimen), and neoadjuvant durvalumab plus chemotherapy followed by adjuvant durvalumab (the AEGEAN regimen) are the available options in several countries. However, treatment response to neoadjuvant or perioperative agents varies among patients, and radiological evaluations sometimes do not correctly reflect tumor status. Therefore, there is a need for clinical examinations that more accurately reflect disease status. Furthermore, precise evaluation of recurrence risk over pathological stage will provide a personalized approach of post-operative care.

Cell-free DNA (cfDNA) are a mixture of small fragmented DNA molecules released from various cells within the body which harbors genetic and/or epigenetic signatures of its cell of origin. Circulating tumor DNA (ctDNA) accounts for a small part of cfDNA (e.g., 0.1–0.003% of cfDNA after surgical resection [6]) that is released into the bloodstream by tumor cells. The detection of ctDNA is a potential groundbreaking biomarker with diagnostic and prognostic value for NSCLC [6,7,8,9] and for some other solid tumors. These ctDNA fragments carry genetic and epigenetic alterations that are characteristic of the tumor, providing a unique molecular fingerprint that can be used to detect targetable driver mutations in patients with metastatic disease as well as to identify and quantify residual tumor cells in patients who have received curative-intent treatments [10,11,12,13].

As a tool to detect minimal residual disease (MRD), several studies reported that positive detection of post-operative ctDNA was a significant poor prognostic factor in patients with surgically resected NSCLC [6,14,15,16,17,18,19,20,21,22,23,24,25,26]. While current ctDNA analysis technology is not sufficiently sensitive to exclude patients who will be cured by surgery alone, some research has suggested the potential utility of ctDNA analysis as a tool to determine patients who will benefit from adjuvant treatment [27,28,29]. In this review, we discuss recent research data on the potential roles of ctDNA analysis in NSCLC patients in this era of perioperative and neoadjuvant chemo-immunotherapy.

## 2. Literature Search

**Figure 1:**
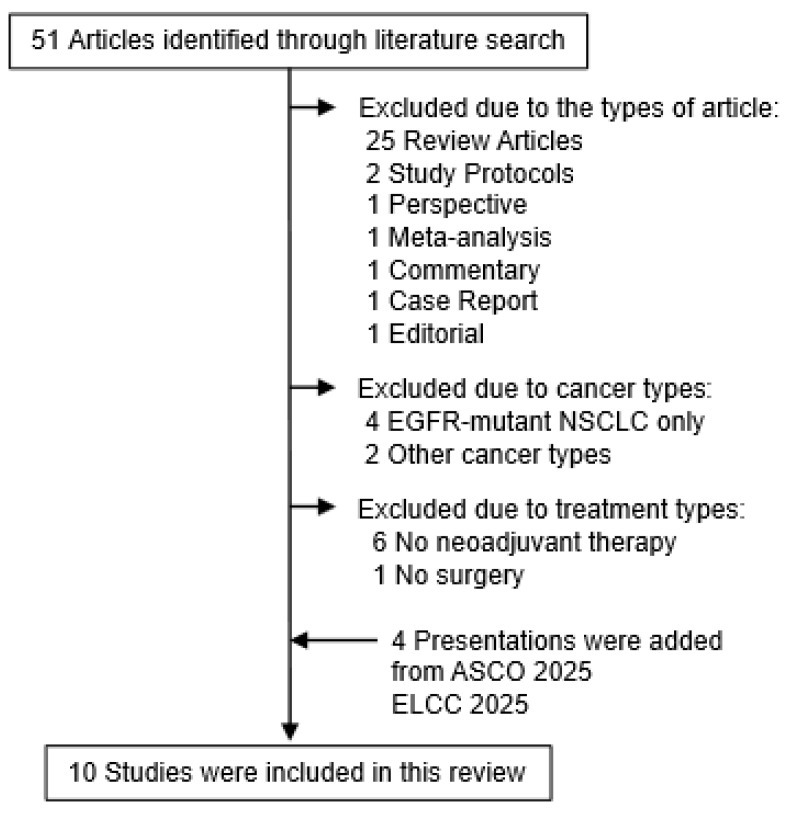
Flowchart of the selection of articles. From the 51 initially identified articles, 45 were excluded because of the reasons summarized in the figure. The 6 remaining articles were selected for the final review. Furthermore, 4 presentations were added from ASCO 2025 [30,31,32] and ELCC 2025 [33]. The total 10 studies were summarized in this review. One ASCO presentation was replaced with the concurrent publication in The New England Journal of Medicine in 2025 [34].

We performed a systematic literature search using PubMed to identify relevant studies published between 1 January 2020 and 31 May 2025. The following keywords were used for the search: lung cancer [all fields] AND circulating tumor DNA [all fields] AND neoadjuvant [all fields]. Of the 51 articles initially identified, 32 were excluded because of article type (25 review articles, 2 study protocols, 1 perspective, 1 meta-analysis, 1 commentary, 1 case report, and 1 editorial). A further 6 studies were excluded because they focused only on NSCLC with epidermal growth factor receptor mutation (*n* = 4) or other cancer types (*n* = 2). Additionally, 7 studies were excluded for lacking neoadjuvant therapy (*n* = 6) or surgical intervention (*n* = 1). Three abstracts at American Society of Clinical Oncology (ASCO) 2025 and one at European Lung Cancer Conference (ELCC) 2025 described the results of ctDNA analysis from phase II or III clinical trials [30,31,32,33], therefore we included these data in this review (Figure 1). The details of these studies are summarized in Table 1. Ongoing clinical trials related to this topic were identified through https://www.clinicaltrials.gov.

## 3. Current Clinical Challenges of Perioperative/Neoadjuvant Chemo-Immunotherapy

While perioperative/neoadjuvant chemo-immunotherapy is now being widely used in clinical practice as a treatment for patients with resectable NSCLC, several important questions remain unresolved.

### 3.1. Need for Neoadjuvant Chemo-Immunotherapy

Some patients with clinical stage II–III disease are curable by surgical resection alone, and the application of neoadjuvant chemo-immunotherapy would be an over-treatment for these patients. Therefore, identifying patients with clinical stage II–III disease who can be treated with surgery alone is critical.

### 3.2. Adequate Treatment Courses of Neoadjuvant Chemo-Immunotherapy

Three cycles of neoadjuvant chemo-immunotherapy were applied in the CheckMate-816 trial and up to four cycles were applied in other studies including the KEYNOTE-671 and AEGEAN trials. However, the number of adequate treatment courses of neoadjuvant chemo-immunotherapy remains unclear. We sometimes experience patients who show a dramatic response to neoadjuvant chemo-immunotherapy after only one course of treatment. Several studies evaluated the association between treatment cycles of neoadjuvant therapy and pathological response or ctDNA results and the results usually suggested that more treatment may be better [41,42,43]. Therefore, it would be possible that the optimal number of treatment cycles of neoadjuvant chemo-immunotherapy may thus vary among individual patients.

### 3.3. Monitoring Tumor Response to Neoadjuvant Treatment

Clinical tumor response during neoadjuvant treatment is currently evaluated by radiological examination, mainly with computed tomography (CT). However, we sometimes observe a discordance between the radiological response and actual (pathological) tumor remission after chemo-immunotherapy, which may be because of the difficulty to distinguish true disease progression and inflammatory responses.

### 3.4. Recurrence Risk Stratification After Pulmonary Resection

As a result of the quite high efficacy of chemo-immunotherapy in some patients with metastatic NSCLC, as evidenced by the so-called “long tail effect” in survival curves, it is hypothesized that some patients may be cured by neoadjuvant treatment alone. These patients may not require adjuvant anti-PD-1/PD-L1 monotherapy and strict radiological follow-up after surgery or may even not require pulmonary resection if we can precisely detect MRD after neoadjuvant treatment. Virtually all clinical trials have shown that pathological evaluation of resected specimens, after neoadjuvant chemo-immunotherapy, was significantly associated with survival outcomes of patients. However, it is also true that some patients experienced disease recurrence despite the diagnosis of pathological complete response (pCR) after neoadjuvant chemo-immunotherapy [34], suggesting that pCR does not always indicate a cure of NSCLC. One possible mechanism for such phenomenon includes that there might be small nests of viable tumor cells in areas that were not covered by the sections. Another possibility includes heterogeneous therapeutic effects between micro-metastatic lesions and primary tumors/metastatic lymph nodes, partially due to the heterogeneity of genetic and/or epigenetic alterations.

On the other hand, for patients with residual disease even after surgical resection, intensive adjuvant treatment, e.g., adding novel therapeutic agents to adjuvant anti-PD-1/PD-L1 monotherapy, may increase the possibility of cure or improve the event-free and overall survival.

## 4. Utility of ctDNA Analysis as a Tool to Solve the Above Clinical Questions

The use of ctDNA as a tool to evaluate tumor burden or detect MRD offers some advantages over traditional radiological examinations. ctDNA analysis is minimally invasive, requiring only a small amount of blood sample (~20 mL), which allows repeated sampling over time. ctDNA can provide a comprehensive view of the tumor’s genetic landscape, capturing heterogeneity or therapy-resistant clones of residual disease [22,42,43,44]. Therefore, ctDNA analysis is emerging as a potential promising biomarker to refine clinical decision-making in the perioperative setting in early-stage NSCLC.

However, at the same time, it should be noted that various techniques are now being developed for ctDNA detection analysis with distinct sensitivities and specificities. Different ctDNA assays may have been used in each clinical trial summarized in this review (Table 1); therefore, it should be noted that the outcomes, e.g., the positive rates and hazard ratios, described in this review may not be generalizable.

### 4.1. Utility of ctDNA Analysis Before and During Neoadjuvant Treatment

In the previous studies of MRD detection in patients who received upfront surgery for NSCLC, compared with post-surgical ctDNA analysis, pre-treatment ctDNA was associated with factors related to DNA shedding [44], such as larger tumor size, clinical stage (tumor burden), and squamous cell histology, and was less associated with patient outcomes [7,14,45]. ctDNA analysis may be beneficial to identify patients with a high tumor burden (candidates for neoadjuvant treatment rather than upfront surgery) or low tumor burden (candidates for limited surgical resection). However, currently, the use of pre-treatment ctDNA analysis for these applications has not been formally explored or demonstrated, and currently pre-treatment ctDNA testing is generally used to assess baseline values.

ctDNA analysis during neoadjuvant treatment may serve as a valuable tool for monitoring therapeutic response. A potential discordance between radiographic and pathologic responses following neoadjuvant chemo-immunotherapy has been reported [46], underscoring the need for more reliable biomarkers for response, such as ctDNA, to accurately evaluate treatment efficacy and guide adequate perioperative management. In radiological examinations, it is sometimes difficult to distinguish between viable tumor areas and fibrotic and/or necrotic ones of the primary lesion during neoadjuvant chemo-immunotherapy. Furthermore, it is even more difficult to distinguish between viable metastatic tumor cells and immune cell response within the regional lymph nodes. On the other hand, the half-life of ctDNA is extremely short, reportedly approximately a half hour; therefore ctDNA can accurately reflect the “viable tumor burden” at that point of examination. Thus, ctDNA analysis is anticipated to provide useful information as to whether neoadjuvant treatment should be continued or if neoadjuvant treatment should be terminated and instead pulmonary resection should be performed because of progressive disease.

### 4.2. Utility of ctDNA Analysis Before Surgical Resection

Surgical strategies can be modified on the basis of the remaining tumor cells after neoadjuvant chemo-immunotherapy. However, the remaining tumor cells are usually assessed by pathological examination of resected tumor specimens. These pathological responses, specifically major pathological response (MPR) and/or pCR, are promising surrogate endpoints for survival after pulmonary resection [47,48,49].

**Figure 2:**
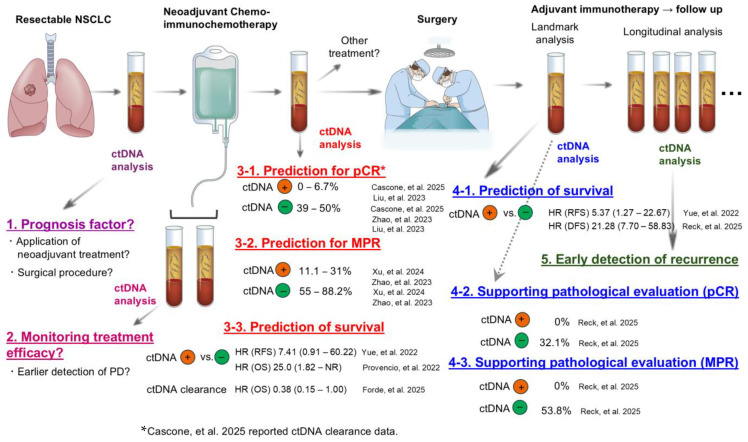
Utility of ctDNA analysis at various time points for patients with NSCLC treated with perioperative chemo-immunotherapy [30,31,32,35,36,38,39,40]. ctDNA⊕ denotes detectable ctDNA in plasma, ctDNA⊖ indicates undetectable ctDNA, and ctDNA clearance means undetectable ctDNA after treatment despite positive ctDNA before treatment. Abbreviations used in this figure: NSCLC: non-small cell lung cancer, ctDNA: circulating tumor DNA, PD: progressive disease, pCR: pathological complete response (the absence of residual invasive cancer in both the primary tumor and lymph nodes), MPR: major pathological response (≤10% residual viable tumor cells), HR: hazard ratio, RFS: recurrence-free survival, OS: overall survival, NR: not reached, Ref.: reference.

Xu et al. reported that ctDNA status before neoadjuvant immunotherapy did not correlate with patient outcome, as described above; however, patients who exhibited ctDNA clearance before surgery had significantly higher MPR rates compared with those with residual ctDNA (88.2% vs. 11.1%, *p* < 0.001) [40]. Other groups also reported the correlation between ctDNA clearance and pCR [31] or MPR [35,37,39]. ctDNA negativity after neoadjuvant chemo-immunotherapy was also shown to be a biomarker to predict pathological response [38]. The detailed results of these studies are summarized in Figure 2 (3-1. Prediction for pCR and 3-2. Prediction for MPR).

Because ctDNA status after neoadjuvant chemo-immunotherapy is associated with pathological response and the pathological response is associated with survival outcome of patients, ctDNA status after neoadjuvant chemo-immunotherapy may reasonably predict patient outcome. Studies have reported that ctDNA negativity and ctDNA clearance were significantly associated with superior recurrence-free and overall survival (3-3. Prediction of survival, Figure 2) [30,36]. For example, Yue et al. reported that detected ctDNA after neoadjuvant therapy tended to be associated with worse recurrence-free survival, with a HR of 7.41 (95% CI: 0.91–60.22, *p* = 0.03) [35].

### 4.3. Landmark ctDNA Status as a Tool for MRD Detection

Evaluation of ctDNA status after surgery is often referred to as “landmark analysis” and the detection of ctDNA at this landmark point is considered MRD positive. Yue et al. performed ctDNA analysis using blood samples obtained between 3 and 8 days post-surgery. The authors found that 31.8% of patients had detectable ctDNA and positive MRD status was an independent risk factor for recurrence (HR, 5.37; 95% CI: 1.27–22.67; log-rank *p* = 0.01) [35]. Another group examined ctDNA status at post-neoadjuvant and post-surgical resection time points and reported that patients who were ctDNA negative at both time points had an 18-month event-free survival rate of 93.8% compared with 47.3% for patients with ctDNA positive status at one of these two time points (HR: 0.15; 95% CI: 0.04–0.94; *p* = 0.005) [38]. In the AEGEAN trial, 168 patients were included in the biomarker-evaluable population, and 10.1% were ctDNA-positive after surgery. Among these MRD-positive patients, 76.5% experienced disease recurrence or death within 12 months postoperatively compared with only 11.9% in the ctDNA-negative group (4-1. Prediction of survival, Figure 2) [32]. Whether ctDNA status at the landmark point will add further prognostic impact over pathological response remains controversial [32,36,37].

### 4.4. Longitudinal ctDNA Analysis for Early Detection of Disease Recurrence

Longitudinal ctDNA analysis is the serial measurement of ctDNA in plasma samples collected at multiple time points after pulmonary resection. The principal advantage of longitudinal ctDNA analysis lies in its capacity to detect molecular recurrence several months prior to radiographic recurrence [14,50]. Even in patients who received neoadjuvant treatment, post-operative longitudinal ctDNA analysis detected disease recurrence an average of 6.83 months earlier than conventional imaging [35].

## 5. Ongoing Clinical Trials Involving ctDNA During Perioperative Chemo-Immunotherapy

In numerous ongoing clinical trials of perioperative immunotherapy for NSCLC, ctDNA analysis is incorporated as part of the primary, secondary or exploratory endpoints. Many of these trials use ctDNA clearance (ctDNA resolution) after neoadjuvant immunotherapy or after surgery for the prediction of pathological response or prognostic classification purposes. Representative trials include NCT05382052 (REAL-NADIM), NCT05778253, NCT06111807, NCT06123754, and NCT06221462 (the PRIORITY trial).

ctDNA status is the primary endpoint in the phase II trial NCT04638582 comparing neoadjuvant pembrolizumab with or without chemotherapy in resectable early-stage NSCLC, which aims to evaluate ctDNA clearance after neoadjuvant immunotherapy and after surgery. The PROPHET trial (NCT06977074) is a phase II proof-of-concept study designed to evaluate the clinical utility of ctDNA testing to optimize the number of induction chemo-immunotherapy cycles in patients with resectable NSCLC. The SAVED LUNG trial (NCT06743555) is a study evaluating the feasibility of omitting surgery in NSCLC patients who achieve a clinical complete response after neoadjuvant therapy; ctDNA-based MRD assessment is also being performed in this trial. In some studies, such as ADOPT-LUNG (NCT06284317) and NCT06902272, the implications of adjuvant treatment in patients who received neoadjuvant treatment and surgical resection are being evaluated with an exploratory analysis of ctDNA status. These trials are summarized in Table 2.

## 6. Conclusions

Promising data have been published from clinical trials regarding the roles of ctDNA analysis in NSCLC treated with neoadjuvant/perioperative chemo-immunotherapy. Many ongoing studies are incorporating ctDNA as a biomarker to predict outcomes and guide treatment decisions. However, it should be noted that the sensitivities of current ctDNA assays are insufficient as tools to detect MRD in early-stage NSCLC; therefore various analytical technologies are currently being developed. Ongoing and future trials, together with the further development of ctDNA detection technologies, will clarify the role of ctDNA analysis in refining perioperative treatment strategies and may ultimately enable individualized care in patients with resectable NSCLC.

## Figures and Tables

**Table 1:** Summary of the clinical studies included in this review.

Ref.	Year	N	Neoadjuvant Regimen	Adjuvant Regimen	ctDNA Assay
[35]	2022	22	ICI + CTx vs. ICI combo vs. CTx	-	Tumor-agnostic
[36]	2022	46	Nivo + CTx	Nivo vs. placebo	Tumor-agnostic
[37]	2023	358	Nivo + CTx vs. CTx	-	Tumor-informed
[38]	2023	52	Nivo + CTx vs. Nivo	-	Tumor-informed
[39]	2023	78	Camrelizumab + apatinib	-	Tumor-agnostic
[40]	2025	45	Sintilimab + CTx	-	Tumor-agnostic
[30]	2025	358	Nivo + CTx vs. CTx	-	Tumor-informed
[31]	2025	461	Nivo + CTx vs. CTx	Nivo vs. placebo	-
[32]	2025	802	Durval + CTx vs. CTx	Durval vs. placebo	Tumor-informed
[33]	2025	86	Nivo + CTx vs. CTx	Nivo vs. placebo	Tumor-agnostic

Abbreviations: Ref.: reference, ICI: Immune checkpoint inhibitor, CTx: Chemotherapy, Nivo: nivolumab, Durval: durvalmab.

**Table 2:** ctDNA application in ongoing NSCLC neoadjuvant clinical studies.

NCT	Primary Endpoints	Secondary Endpoints	Exploratory Analysis
NCT04638582	ctDNA clearance (pre/post-surgery)	MPR, pCR, radiologic response	Correlation of ctDNA dynamics with DFS/OS
NCT05382052	Association of ctDNA clearance with PFS	DFS, OS	Longitudinal ctDNA monitoring feasibility
NCT05778253	ctDNA clearance, AI-based prediction of pCR	MPR, ORR, DFS, OS, QoL, surgical outcomes	Correlation of AI pathology with clinical outcomes
NCT06111807	Sensitivity/specificity of ctDNA assays	Association of ctDNA with recurrence/DFS	ctDNA-guided risk stratification
NCT06123754	MPR (pre-op), EFS (post-op)	pCR, DFS, OS, safety	Biomarker analysis, ctDNA dynamics
NCT06221462	pCR/MPR, safety, surgical feasibility	DFS, OS, AEs	ctDNA clearance, radiologic-pathologic correlation
NCT06284317	DFS in non-pCR patients	DFS/OS in pCR group, safety, ctDNA analysis	Correlation of ctDNA with recurrence and survival
NCT06743555	Feasibility and safety of surgery omission	EFS, OS, recurrence rate	ctDNA monitoring for recurrence detection
NCT06902272	Correlation between ctDNA and pCR/MPR	DFS, OS, ctDNA kinetics	ctDNA MRD detection, longitudinal profiling
NCT06977074	pCR, MPR, surgical eligibility	DFS, OS, AEs	ctDNA-guided treatment optimization

Abbreviations: ctDNA: circulating tumor DNA, pCR: pathological complete response, MPR: major pathological response, DFS: disease-free survival, OS: overall survival, EFS: event-free survival, PFS: progression-free survival, ORR: objective response rate, QoL: quality of life, AEs: adverse events, AI: artificial intelligence.

## Data Availability

This review article summarized published data and the data that support the findings of this study are available from the corresponding author (K.S.) upon reasonable request.

## Abbreviations

The following abbreviations are used in this manuscript:

NSCLC: Non-small cell lung cancer, ctDNA: Circulating tumor DNA, MRD: Minimal residual disease, ASCO: American Society of Clinical Oncology, ELCC: European Lung Cancer Conference, MPR: Major pathological response, pCR: Pathological complete response, CT: Computed tomography, FDG-PET/CT: Fluorodeoxyglucose–positron emission tomography/computed tomography, PD-1: Programmed cell death protein 1, PD-L1: Programmed death-ligand 1, HR: Hazard ratio, RFS: Recurrence-free survival.
